# Testing the Disgust-Based Mechanism of Homonegative Attitudes in the Context of the COVID-19 Pandemic

**DOI:** 10.3389/fpsyg.2021.647881

**Published:** 2021-05-17

**Authors:** Aleksandra Szymkow, Natalia Frankowska, Katarzyna Galasinska

**Affiliations:** Center for Research on Biological Basis of Social Behavior, SWPS University of Social Sciences and Humanities, Warsaw, Poland

**Keywords:** behavioral immune system, disgust, homonegativity, gay, lesbian, COVID-19

## Abstract

Negative attitudes and stigmatization can originate from the perception of a disease-related threat. Following the spread of the COVID-19 pandemic, it is often suggested that incidents of discriminatory behavior are the result of defense mechanisms aimed at avoiding pathogens. According to the behavioral immune system theory, people are motivated to distance themselves from individuals who show signs of infection, or who are only heuristically associated with a disease, primarily because of the disgust they evoke. In this paper we focus on negative attitudes toward gay men and lesbians who are among social groups that have been persistently framed as “unclean.” In our correlational study (*N* = 500 heterosexual participants; Polish sample data collected during the first COVID-19 lockdown in Poland, in March/April 2020) we tested moderation models derived from the behavioral immune system theory. Specifically, we investigated whether perceived vulnerability to disease and perceived threat of contracting COVID-19 moderate the relation between disgust and homonegativity. We found that sexual disgust (but not pathogen nor moral disgust) predicted homonegative attitudes. This effect was stronger for participants expressing higher levels of perceived vulnerability to disease but was not dependent on the perception of the COVID-19 threat. The results reaffirm previous evidence indicating a pivotal role of disgust in disease-avoidance mechanisms. They also point to functional flexibility of the behavioral immune system by demonstrating the moderating role of perceived vulnerability to disease in shaping homonegative attitudes. Finally, they show that the threat of COVID-19 does not strengthen the relationship between disgust and homonegativity.

## Introduction

The COVID-19 pandemic has set the ground for testing disease-related mechanisms of various social phenomena in real pathogen-threat conditions. In this context, one of the highly relevant domains concerns social attitudes, as they often have disease-related origins (Faulkner et al., 2004). Here, we focus on homosexual individuals, both men and women, who have been frequently framed as “unclean,” and associated with germs and diseases (such as AIDS) (Crandall et al., 1997; Herek, 2002; Greene and Banerjee, 2006; Golec de Zavala et al., 2014; Pachankis et al., 2015; Filip-Crawford and Neuberg, 2016).

One of the most relevant theoretical frameworks applied to explain negative social attitudes such as prejudice, ethnocentrism, or homonegativity, is the evolution-based theory of the behavioral immune system (BIS; Curtis et al., 2011; Murray and Schaller, 2016; Ackerman et al., 2018). As mobilizing the physiological immune system is metabolically costly (Conner and Grisham, 1996), it was suggested that another set of psychological defense mechanisms designed to mitigate the threat of disease before infection occurs should have evolved (Schaller and Park, 2011). Indeed, the behavioral immune system operates by changing cognition, affect, and behavior in ways that promote pathogen avoidance (Ackerman et al., 2018). The initial detection of threat-relevant cues in the environment triggers affective reactions, leading to adaptive behavior. However, as distinct cues imply different risks, behavioral immune system exhibits functional flexibility to both individual (e.g., perceived self-infectability) and contextual (e.g., a pandemic) factors (Ackerman et al., 2018). For instance, temporarily heightened vulnerability to disease amplifies negative reactions to foreigners, particularly of nationalities less familiar to the participants (Faulkner et al., 2004). Additionally, vaccinated participants tend to express less prejudice toward immigrants than do unvaccinated participants (Huang et al., 2011). The BIS is also prone to overgeneralization. Similarly to a smoke detector, it sometimes becomes activated even in the absence of a real disease threat. Thus, we can observe avoidance tendencies triggered by the presence of non-infectious physical and mental abnormalities, such as disfigurements, disabilities, or obesity (Park et al., 2003; see also Nussinson et al., 2018).

Researchers point to disgust as the fundamental affective factor underlying pathogen avoidance mechanisms, because it functions as the first line of defense against infection (see Oaten et al., 2009, for a review). Disgust triggers reactions directed to minimize disease risk after we have encountered the disease threat, motivating us to avoid contaminated sources before we come into contact with them (Rubio-Godoy et al., 2007). To date, there is a large body of empirical data demonstrating the crucial role of disgust and disgust sensitivity in shaping social attitudes and behaviors. Disgust facilitates out-group dehumanization (Buckels and Trapnell, 2013) and xenophobia (Zakrzewska et al., 2019), as well as prejudicial reactions to individuals not being actually diseased but only heuristically associated with the presence of diseases (e.g., obese or disfigured people; Harvey et al., 2002). It has recently been suggested that the behavioral immune system uses out-group membership as a cue for infectiousness (Bressan, 2020).

Nussbaum (2001) claimed that homosexuality frequently appears as an object of disgust. Indeed, a substantial body of research points to the relationship between disgust and homonegative attitudes (Herek, 1993). For instance, manipulating disgust by exposing participants to a noxious ambient odor amplifies negative attitudes toward sexual minorities (Inbar et al., 2012). Additionally, Inbar et al. (2009) demonstrated that individuals high in disgust sensitivity perceived gay people more negatively than did those low in disgust sensitivity. What seems to be the crucial point is that the observed impact of disgust on attitudes tends to be larger in response to gay men than lesbians (Inbar et al., 2012; Kiss et al., 2020). The reason for that could be that gay men’s engagement in anal intercourse is perceived as unhygienic (Kiss et al., 2020) and that male homosexuality is associated with germs and diseases such as AIDS (Crandall et al., 1997; Pachankis et al., 2015). Bettinsoli et al. (2019) showed that across 23 countries, gay men were disliked more than lesbians. Therefore, it is strongly recommended to analyze data on attitudes toward gay men and lesbians separately (Kiss et al., 2020).

Another key issue relates to the multifaceted nature of disgust. Despite the common ground in the form of the affective state of repulsion, different facets of disgust (i.e., pathogen, moral, and sexual) are considered to be designed by natural selection to solve different functional problems (Tybur et al., 2009). Pathogen disgust motivates the avoidance of diseases and is likely to be elicited when a pathogenic threat is salient. Sexual disgust motivates the avoidance of sexual partners and behaviors that can lead to negative biological repercussions (e.g., sex with genetic relatives). However, it has also been suggested that sexual stimuli and behaviors that transgress culturally normative conceptualizations of sexuality may trigger sexual disgust (Morrison et al., 2019). Likewise, it should be indicated that perceived vulnerability to disease (PVD; Duncan et al., 2009) correlates positively with pathogen disgust and sexual disgust (Tybur et al., 2009). This is understandable, as there are substantial disease risks associated with sexual behavior. Moral disgust, the final domain of disgust, drives the avoidance of social norm violators and is distinct from the other two domains (Tybur et al., 2009).

The evidence identifying the relationship between specific disgust domains and homonegativity is highly inconsistent. The vast majority of studies demonstrating that disgust sensitivity predicts an increased level of homonegative attitudes focused primarily on only one domain of this phenomenon, namely, pathogen disgust (Haidt et al., 1994; Inbar et al., 2009, 2012). Some studies that relied on the more sophisticated scale of three domains of disgust (TDDS; Tybur et al., 2009) showed stronger associations between homonegative attitudes and sexual disgust than pathogen or moral disgust (Ray and Parkhill, 2020). However, some research indicated that there are no associations at all (Smith, 2012). Specifically, Smith (2012) manipulated pathogen and moral disgust (but not sexual disgust) and found no causal relationship between these two disgust types, and implicit and explicit anti-gay attitudes. Taking all these insights into account, it seems that it is still an open question as to which type of disgust, if any, is the most prominent predictor of homonegativity (see also Smith, 2012; Ray and Parkhill, 2020).

Our study focused on three domains of disgust as potential predictors of homonegative attitudes in the context of the COVID-19 pandemic. On the basis of the BIS theory, we hypothesized that pathogen and sexual disgust would predict negative attitudes toward gay persons, and that this effect would be stronger for gay men than for lesbians. It could be argued that sexual disgust is not directly related to pathogen threats and, as such, cannot be derived from the theory of BIS as a predictor of social attitudes (Ray and Parkhill, 2020). We want to make a different argument that, since certain pathogens can be transmitted sexually (e.g., through anal intercourse), sexual disgust becomes an important indicator of BIS activation. Thus, we expect sexual disgust to be a stronger predictor of homonegative attitudes than pathogen disgust.

Moreover, we predict that the above effects will be moderated by concerns about disease. As the assumptions of BIS theory (e.g., Schaller and Park, 2011) and previous research suggest, worrying about contracting an illness can significantly predict social attitudes (such as prejudice). This anxiety can have various origins—it can result from individual differences (e.g., perceiving one’s vulnerability to disease) and can also depend on external circumstances (e.g., temporarily activated salience of pathogens). Thus, we predict that perceived vulnerability to disease will moderate the effect. Specifically, we predict that individuals who express higher levels of PVD will show a stronger connection between disgust and homonegativity than those expressing lower levels of this variable. Furthermore, we wanted to investigate whether the relation between disgust and homonegativity is moderated by the perceived threat of contracting COVID-19. As Faulkner et al. (2004) noticed, any contextual information that implies increased vulnerability to infection may amplify negative reactions to categories of people heuristically associated with a disease. What follows is that a specific psychological reaction (such as disgust or perceived vulnerability to illness) may be associated with the perceived threat of disease in a given situation. Indeed, a temporary salience of disease amplifies xenophobic attitudes (Faulkner et al., 2004), similarly to disease salience decreasing the desire to affiliate with out-group members (Millar et al., 2020). Importantly, it has been shown that activating COVID-19 concerns (by reading about mortality statistics and government lifestyle regulations) results in higher germ aversion among participants, compared to the control condition (Bacon and Corr, 2020). Stevenson et al. (2021) also indicated that students showed higher levels of core disgust and germ aversion during the COVID-19 lockdown in Australia than before the COVID-19 pandemic. Similarly, Polish women reported higher levels of disgust sensitivity during the COVID-19 pandemic than during the pre-pandemic period (Miłkowska et al., 2021). In addition, Shook et al. (2020) showed that those who were higher in germ aversion and pathogen disgust sensitivity were more concerned with COVID-19 and expressed more preventative behaviors. On the other hand, recent reports indicating the increase of prejudice against Chinese individuals (for more details see: Ackerman et al., 2021), or attacks against the LGBT community in Poland^1^ should also be addressed. It is suggested that these negative reactions may be heightened by the pandemic, which may be interpreted as evidence supporting the BIS theory. However, this may not necessarily be the case. What should be highlighted here is that a pandemic is not a type of event that occurs frequently over a course of a human lifetime. Historically it has also been a rare phenomenon, due to multiple features of ancestral societies, such as relatively small-scale geographic mobility and limited routes of disease transmissions. For this reason, it is unlikely that defense mechanisms specific to pandemics have evolved among humans (Ackerman et al., 2021; see also Varella et al., 2021).

Taking these considerations into account, we investigated whether the threat of COVID-19 plays any significance in moderating the BIS mechanism. Specifically, we tested whether a higher level of the perceived threat of COVID-19 would be associated with a stronger relation between disgust sensitivity and homonegativity.

## Materials and Methods

### Participants

We recruited Polish respondents from the SWPS University of Social Sciences and Humanities (through the Sona system) and a social media web page^2^. The final sample consisted of 588 participants who agreed to participate in the study on social attitudes and complete a web-based survey through Qualtrics. We conducted a frequency analysis, which revealed that 60 participants reported being bisexual, 24 reported being gay, and 4 participants chose the “other” option regarding their sexual orientation. For this study, we analyzed data for participants who identified as heterosexual: 500 respondents (421 women and 79 men; *M*_*age*_ = 20.18, *SD* = 9.20). Sensitivity calculations made using the G^∗^Power 3.1.9.2 software (Faul et al., 2007) indicate that for a model including one tested predictor and three total predictors, the *N* = 500 sample is sufficient to detect an effect at a size of *f* > 0.016 (*R*^2^ > 0.0157) with 1-Beta > 0.8. Given that the observed *R*^2^ for all significant moderations is > 0.2, our study has sufficient power. Data collection started on March 18, 2020, after the government announced the first COVID-19 pandemic lockdown in Poland, and ended on April 9, 2020.

### Procedure and Materials

#### Disgust Propensity

To measure disgust propensity, we used the Three Domains of Disgust Scale (TDDS; Tybur et al., 2009). The questionnaire describes 21 situations that refer to three disgust domains: *pathogen disgust* (e.g., “Standing close to a person who has body odor”), *sexual disgust* (e.g., “Performing oral sex”), and *moral disgust* (e.g., “Students cheating to get good grades”). Participants used a 7-point scale (from 1 = *it is not disgusting at all* to 7 = *it is extremely disgusting*) to rate each situation. We averaged scores for the three subscales separately: moral disgust (*α* = 0.81), pathogen disgust (*α* = 0.70), and sexual disgust (*α* = 0.76).

#### Homonegativity

We used the Modern Homonegativity Scale (MHS; Morrison and Morrison, 2002) to separately measure attitudes toward gay men (MHS-G; *α* = 0.92) and lesbians (MHS-L; *α* = 0.93). Each scale consists of 12 statements that participants rated from 1 = *strongly agree* to 5 = *strongly disagree*. We also created a general index of homonegativity by averaging the results about lesbians and gay men (MHS; *α* = 0.97).

#### Perceived Vulnerability to Disease

To measure subjective perceptions of susceptibility to disease, we used the PVD scale (Duncan et al., 2009). The scale is composed of two subscales: perceived infectability (PI; 7 items; *α* = 0.80) and germ aversion (GA; 8 items; *α* = 0.71). The perceived infectability subscale refers to one’s beliefs about their susceptibility to infectious diseases. In comparison, the germ aversion subscale assesses emotional discomfort in contexts involving a high risk of pathogen transmission. The scale consists of 15 statements rated by respondents on a 7-point scale (from 1 = *strongly disagree* to 7 = *strongly agree*). Some researchers investigating the role of perceived vulnerability to disease analyzed the general index of PVD (Faulkner et al., 2004), while others analyzed the subscales separately (Smith, 2012; Brown and Sacco, 2020). As the subscales emphasize different aspects of perceived vulnerability (germ aversion is more emotion-based, while perceived infectability is more cognitive-based), we decided to investigate the role of GA and PI separately.

#### Perceived Threat of COVID-19

To measure the perceived threat of COVID-19, participants indicated their agreement with eight statements on a 7-point scale (from 1 = *strongly disagree* to 7 = *strongly agree*). The scale included items measuring the extent to which participants perceived coronavirus to be a threat to their lives (e.g., “I think that coronavirus is a real threat to my life” and “I am convinced that the media are exaggerating the threat of coronavirus”). Ratings for these items were averaged to form an index of the perceived threat of COVID-19 (*α* = 0.71)^3^.

## Results

We first calculated descriptive statistics for all variables and examined Pearson’s correlations between all variables using IBM SPSS Statistics 25. We adjusted threshold levels of significance for correlation coefficients due to Bonferroni correction. In the next step, we conducted multiple moderation analyses using Model 1 PROCESS (Hayes, 2013).

### Initial Analyses

Descriptive statistics and correlation coefficients between continuous variables are presented in Table 1. To maintain the type I error rate at approximately 5% and reduce the probability of this error occurring in multiple testing, we decided to use Bonferroni correction to adjust the level of statistical significance of correlation coefficients (Curtin and Schulz, 1998). For this purpose, we divided the critical level of significance (0.05) by the number of tests performed (36), which resulted in an adjusted level of significance α = 0.001. We found that pathogen disgust positively correlated with sexual disgust (*r* = 0.28, *p* < 0.001) but not with moral disgust (*r* = 0.14, *p* = 0.002). Pathogen disgust was positively correlated with GA (*r* = 0.38, *p* < 0.001) but not with PI (*r* = 0.11, *p* = 0.011), and positively correlated with the perceived threat of COVID-19 (*r* = 0.18, *p* < 0.001). Sexual disgust positively correlated with GA (*r* = 0.21, *p* < 0.001) and with homonegativity toward gays (*r* = 0.28, *p* < 0.001), lesbians (*r* = 0.28, *p* < 0.001), and general homonegativity (*r* = 0.28, *p* < 0.001). The perceived threat of COVID-19 positively correlated with PI (*r* = 0.56, *p* < 0.001) and with GA (*r* = 0.32, *p* < 0.001). Two dimensions of PVD were positively correlated (*r* = 0.28, *p* < 0.001), as were homonegativity indexes toward gays and lesbians (*r* = 0.94, *p* < 0.001).

**TABLE 1:** Means, standard deviations, and correlations among study variables (*N* = 500).

**Variables**	***M***	***SD***	**1**	**2**	**3**	**4**	**5**	**6**	**7**	**8**	**9**
1. Pathogen disgust	5.21	0.84	–								
2. Sexual disgust	3.54	1.08	0.28*	–							
3. Moral disgust	5.22	0.96	0.14	0.13	–						
4. Perceived threat of COVID-19	4.12	0.86	0.18*	0.19	0.04	–					
5. Perceived infectability	3.79	1.04	0.11	0.01*	0.02	0.56*	–				
6. Germ aversion	4.46	0.99	0.38*	0.21*	–0.01	0.32*	0.28*	–			
7. MHS-gays	2.79	0.83	0.02	0.28*	0.06	–0.13	–0.14	0.05	–		
8. MHS-lesbians	2.77	0.84	0.05	0.28*	0.07	–0.12	–0.13	0.05	0.94*	–	
9. General homonegativity (MHS)	2.78	0.82	0.03	0.28*	0.07	–0.13	–0.14	0.05	0.99*	0.99*	–

Cell entries are zero-order Pearson correlation coefficients adjusted due to Bonferroni correction of the p-value, *p < 0.001.

### Moderation Analyses: PVD as a Moderator

First, we tested whether the two subscales of the PVD scale (PI and GA) moderated the positive relation between disgust sensitivity and negative attitudes separately toward gay men and lesbians. As the only domain of disgust that significantly predicted homonegativity was sexual disgust, we present all the analyses exclusively for that kind of disgust. The results of analyses conducted for pathogen and moral disgust are presented in Supplementary Tables 1–4. All moderation analyses were conducted using Model 1 PROCESS (Hayes, 2013) with sex, education, and age as controlled variables^4^. We calculated age as a continuous variable and education as a 3-categorical ordinal variable: primary, secondary, and higher education.

#### The Moderating Role of Perceived Infectability in the Relationship Between Sexual Disgust and Negative Attitudes Toward Gay Men

**FIGURE 1:**
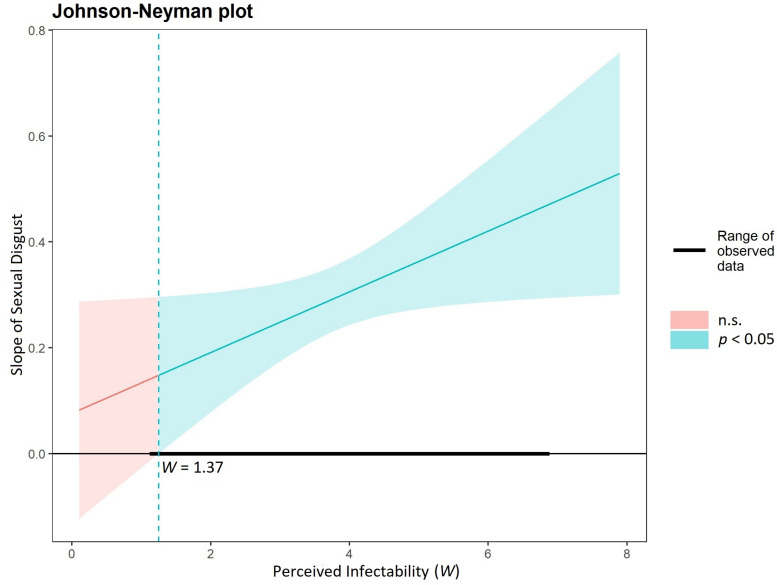
Regions of significance for the conditional effect of sexual disgust presented for perceived infectability as a moderator and negativity toward gay men as the outcome variable.

In the first analysis, we introduced sexual disgust as a predictor, negative attitudes toward gay men as the outcome variable, and PI as the moderating variable. The model was significant, *F*_(6, 489)_ = 24.60, *p* < 0.001, *R*^2^ = 0.23, just as the expected moderation was: *b* = 0.06; 95% *CI* = [0.008, 0.114]. We probed this interaction by using the Johnson-Neyman technique (Hayes and Matthes, 2009), which allowed us to identify the regions of significance for the conditional effect of sexual disgust. As shown in Figure 1, when PI was lower than 1.37, the predicted relation between sexual disgust and negative attitudes toward gay men was not salient. However, starting from the 1.37 point, the higher the PI level, the stronger the relation between sexual disgust and homonegativity toward gay men, which is consistent with our hypotheses. The simple slopes analysis revealed that low, moderate, and high values of PI positively predicted attitudes toward gay men: the coefficient on 1 *SD* below the mean was 2.73, *b* = 0.22; 95% *CI* = [0.141, 0.305], on the mean was 3.77, *b* = 0.29; 95% *CI* = [0.226, 0.348], and on 1 *SD* above the mean was 4.81, *b* = 0.35; 95% *CI* = [0.268, 0.432]. Detailed results are presented in Supplementary Table 5.

#### The Moderating Role of Perceived Infectability in the Relationship Between Sexual Disgust and Negative Attitudes Toward Lesbians

**FIGURE 2:**
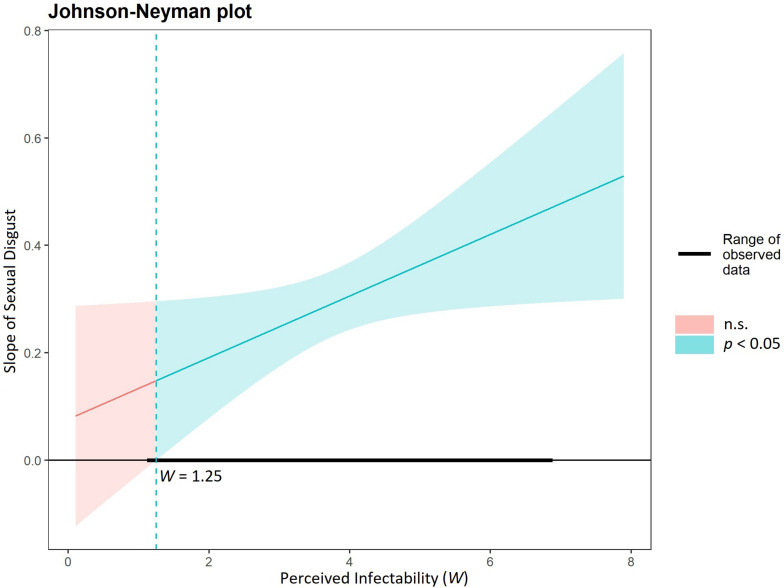
Regions of significance for the conditional effect of sexual disgust presented for perceived infectability as a moderator and negativity toward lesbians as the outcome variable.

The complementary analysis for negative attitudes toward lesbians as the outcome variable, sexual disgust as a predictor, and PI as the moderating variable, revealed that the model was significant, *F*_(6, 489)_ = 23.42, *p* < 0.001, *R*^2^ = 0.22, as was the moderation effect, *b* = 0.06; 95% *CI* = [0.003, 0.110]. As we present in Figure 2, when PI was lower than 1.25, there was no predicted relationship between sexual disgust and negative attitudes toward lesbians. Starting from 1.25 points, the higher the PI level, the stronger the relationship between sexual disgust and negative attitudes toward lesbians. The simple slopes analysis revealed that low, moderate, and high values of PI positively predicted negative attitudes toward lesbians: the coefficient 1 *SD* below the mean was 2.73, *b* = 0.23; 95% *CI* = [0.150, 0.317], at the mean was 3.77, *b* = 0.29; 95% *CI* = [0.230, 0.354], and 1 *SD* above the mean was 4.81, *b* = 0.35; 95% *CI* = [0.268, 0.435]. Detailed results are presented in Supplementary Table 6.

#### The Moderating Role of Germ Aversion in the Relationship Between Sexual Disgust and Negative Attitudes Toward Gay Men

To test the other facet of PVD, we introduced GA as the moderating variable, sexual disgust as a predictor, and negative attitudes toward gay men as the outcome variable. The model was significant, *F*_(6, 489)_ = 21.53, *p* < 0.001, *R*^2^ = 0.21, but we found no moderation effect, *b* = 0.04; 95% *CI* = [-0.021, 0.094]. Detailed results are presented in Supplementary Table 5.

#### The Moderating Role of Germ Aversion in the Relationship Between Sexual Disgust and Negative Attitudes Toward Lesbians

We provided the complementary analysis for negative attitudes toward lesbians as the outcome variable, sexual disgust as a predictor, and GA as a moderator. The model was significant, *F*_(6, 489)_ = 20.73, *p* < 0.001, *R*^2^ = 0.20; however, we found no moderation effect, *b* = 0.03; 95% *CI* = [-0.025, 0.092]. Detailed results are presented in Supplementary Table 6.

### Moderation Analyses: The Perceived Threat of COVID-19 as a Moderator

The next set of moderation analyses introduced the perceived threat of COVID-19 as the moderator in the relationship between sexual disgust and homonegativity. Again, all presented moderation analyses were conducted using Model 1 PROCESS (Hayes, 2013) with sex, education, and age as controlled variables.

#### The Moderating Role of the Perceived Threat of COVID-19 in the Relationship Between Sexual Disgust and Negative Attitudes Toward Gay Men

We conducted this analysis using sexual disgust as a predictor, attitudes toward gay men as the outcome variable, and perceived threat of COVID-19 as a moderator. The model was significant, *F*_(6, 577)_ = 21.41, *p* < 0.001, *R*^2^ = 0.18; however, we found no moderation effect, *b* = 0.04; 95% *CI* = [-0.019, 0.102]. Detailed results are presented in Supplementary Table 5.

#### The Moderating Role of the Perceived Threat of COVID-19 in the Relationship Between Sexual Disgust and Negative Attitudes Toward Lesbians

The complementary analysis was conducted for lesbians with homonegativity as the outcome variable, sexual disgust as a predictor, and perceived threat of COVID-19 as a moderator. The model was significant, *F*_(6, 577)_ = 20.35, *p* < 0.001, *R*^2^ = 0.18, but again, we found no moderation effect, *b* = 0.04; 95% *CI* = [-0.017, 0.106]. Detailed results are presented in Supplementary Table 6.

## Discussion

Disease avoidance mechanisms and their potential role in explaining social attitudes have recently received much scientific attention (e.g., Mentser and Nussinson, 2020; Kramer and Bressan, 2021). In the context of the ongoing COVID-19 pandemic, they have become even more pertinent (Bressan, 2020; Seitz et al., 2020; Hromatko et al., 2021). Our study was designed to test the predictions emerging from the behavioral immune system theory (Ackerman et al., 2018). Specifically, we tested whether pathogen and sexual (but not moral) disgust would predict negative attitudes toward gay men and lesbians, and whether perceived vulnerability to disease (i.e., perceived infectability, germ aversion) and perceived threat of COVID-19 moderated these predicted associations.

Our results point to the significance of sexual disgust in predicting homonegative attitudes. The more participants declared experiencing sexual disgust, the more negative attitudes they held toward gay men and lesbians. Surprisingly, we did not notice any effects to be stronger for gay men than lesbians. Additionally, along with our hypotheses, moral disgust did not play any role in predicting homonegative attitudes. However, contrary to our expectations, pathogen disgust did not predict homonegativity either. Although we predicted that the relationship between pathogen disgust and homonegativity would not be as strong as in the case of sexual disgust, pathogen disgust produced no effects at all. Such results resonate with the data demonstrated by Smith (2012), and Ray and Parkhill (2020).

Ray and Parkhill (2020) showed that the relationship between heteronormativity and hostility toward gay men was mediated by sexual disgust but not pathogen or moral disgust. Importantly, they argued that the null effects for pathogen disgust eliminate the disease-avoidant approaches (i.e., BIS) as reasonable frameworks for explaining hostility toward gay men. In our view, such a conclusion would be warranted only if we could be absolutely certain that sexual disgust is not associated with pathogen avoidance. However, although pathogen disgust and sexual disgust proved their undeniable distinctiveness, they also showed some similarities in terms of pathogen avoidance (Tybur et al., 2009). Specifically, both pathogen and sexual disgust showed positive and equally strong relationships with PVD, indicating that each of them can motivate disease-avoidant behaviors. This was predicted by the authors of TDDS (Tybur et al., 2009), who considered the potential disease risk associated with sex. Taking these findings into account, our results would suggest that sexual disgust is the most likely determinant of homonegative attitudes, however it may also carry a disease-avoidant function. This points to the specificity of the disgust-driven mechanisms. As gay persons can be viewed in light of their sexual behavior and associated with sexually transmitted diseases (Pachankis et al., 2015), it is primarily sexual disgust that may prominently drive attitudes toward them, rather than pathogen disgust.

Our data partly confirmed the moderating role of perceived vulnerability to disease in the relationship between sexual disgust and negative attitudes toward gay men and lesbians. We observed the expected effects for the perceived infectability subscale but not for the germ aversion subscale of PVD. Specifically, the more participants perceived themselves as susceptible to diseases, the stronger was the association between sexual disgust and negative attitudes for both gay men and lesbians. This supports the idea of the behavioral immune system and its flexibility: the system’s response differs depending on the individual’s perceived vulnerability to disease (Ackerman et al., 2018). However, it is unclear why the results were significant only for the cognition-based facet of the PVD, and not for the germ aversion subscale. Perhaps, just as in the case of pathogen disgust measured by TDDS, the items of the GA subscale do not cover aversion toward sexually transmitted pathogens (examples of items: “It truly bothers me when people sneeze without covering their mouths,” “I do not like to write with a pencil someone else has obviously chewed on,” “I prefer to wash my hands pretty soon after shaking someone’s hand”). On the other hand, the PI subscale refers to a rather general vulnerability to wide classes of diseases.

Analyses for the moderating role of the perceived threat of COVID-19 showed that this kind of threat played no role in the relationship between sexual disgust and homonegativity. Situational circumstances, such as a pandemic, are thought to strengthen the potential effects of disease-avoidance mechanisms (e.g., Bacon and Corr, 2020; Millar et al., 2020; Sorokowski et al., 2020; Hromatko et al., 2021; Miłkowska et al., 2021; Stevenson et al., 2021). Our research indicates, however, that concerns about COVID-19 have no effect on attitudes toward gay persons. The perceived likelihood of becoming infected with this illness did not strengthen the relationship between sexual disgust and homonegativity. It could be argued that such an effect proves the flexible functionality of the BIS. When a given pathogen (e.g., COVID-19) is transmitted in a specific way (e.g., through respiratory droplets), the threat of becoming infected should motivate avoidance of people and situations that increase the risk of this particular contact (e.g., standing near coughing people). If gay persons are associated with sexually transmitted diseases (e.g., AIDS; Crandall et al., 1997; Pachankis et al., 2015), then there is no reason to expect that the threat of COVID-19 would motivate avoidance of gay men or lesbians. If we focused on the threat of AIDS, however, then we could expect that motivation.

To conclude, our short report provides some evidence confirming the predictions stemming from the behavioral immune system theory in predicting homonegative attitudes. It reaffirms the previous evidence indicating the pivotal role of disgust in disease-avoidance mechanisms, and specifying sexual disgust as the most important aspect when considering attitudes toward gay men and lesbians. It also emphasizes the functional flexibility of the BIS by demonstrating the moderating role of perceived vulnerability to disease. Finally, it shows for the first time that the threat of COVID-19 does not strengthen the relationship between disgust and homonegativity.

## Data Availability Statement

The datasets presented in this study can be found in online repositories. The names of the repository/repositories and accession number(s) can be found below: Open Science Framework: https://osf.io/976qp.

## Ethics Statement

The studies involving human participants were reviewed and approved by The Ethical Review Board at SWPS University of Social Sciences and Humanities, Faculty of Psychology in Sopot. Written informed consent for participation was not required for this study in accordance with the national legislation and the institutional requirements.

## Author Contributions

AS and NF conceptualized and designed the research. NF conducted the research. AS analyzed and interpreted the data. AS, NF, and KG wrote the manuscript. All authors contributed to the article and approved the submitted version.

## Conflict of Interest

The authors declare that the research was conducted in the absence of any commercial or financial relationships that could be construed as a potential conflict of interest.

## Footnotes

1 https://www.reuters.com/article/us-poland-lgbt-europe-trfn-idUSKBN2AA20S

2 www.facebook.pl

3 We also had additional measures in the study (political beliefs, self-perceived religiosity, subjective well-being, agreement with the government’s decisions regarding COVID-19, sense of control, system justification, social distancing toward out-groups, pregnancy, gestational age, fertility, last flu vaccination, education level, and earnings). We do not discuss them, as they served to verify hypotheses not related to the topic presented here.

4 These variables should be controlled if not taken into consideration (see for example Oaten et al., 2009; Ackerman et al., 2018). We provide the description of the effects of covariates in Supplementary Material.
