# Supplementary material for: Testing the Disgust-Based Mechanism of Homonegative Attitudes in the Context of the COVID-19 Pandemic
Source: Front Psychol. 2021 May 17;12:647881. doi: 10.3389/fpsyg.2021.647881 (PMC8165159; doi:10.3389/fpsyg.2021.647881)
Supplement: Supplementary file 1 [file Data_Sheet_1.PDF]

*Supplementary Material*

Table 1

*Indirect effects and confidence intervals of the Moderation Analysis of the relationship between Pathogen Disgust and homonegativity towards gay men moderated by Perceived Infectability, Germ Aversion and Perceived Threat of Covid-19*

| | Gay men homonegativity | | | | | |
|---|---|---|---|---|---|---|
| | | | | | 95% CI* | |
| | *b* | *SE* | *t* | *p* | *LL* | *UL* |
| **Pathogen Disgust (X)** | 0.10 | 0.17 | 0.59 | 0.559 | -0.235 | 0.433 |
| **Perceived Infectability (W)** | -0.02 | 0.24 | -0.07 | 0.944 | -0.480 | 0.447 |
| **PathogenDisgust × PI (XW)** | -0.01 | 0.04 | -0.32 | 0.750 | -0.099 | 0.072 |
| **Sex** | 0.54 | 0.10 | 5.52 | < 0.001 | 0.349 | 0.734 |
| **Age** | -0.00 | 0.00 | -0.84 | 0.401 | -0.013 | 0.005 |
| **Education** | 0.27 | 0.09 | 3.06 | 0.002 | 0.096 | 0.440 |
| | $F(6, 489) = 8.25$, $p < 0.001$, $R^2 = 0.09$ | | | | | |
| **Pathogen Disgust (X)** | 0.04 | 0.18 | 0.23 | 0.820 | -0.657 | 3.139 |
| **Germ Aversion (W)** | 0.09 | 0.21 | 0.42 | 0.672 | -0.329 | 0.510 |
| **Pathogen Disgust × GA (XW)** | -0.01 | 0.04 | -0.18 | 0.622 | -0.086 | 0.071 |
| **Sex** | 0.59 | 0.10 | 6.02 | < 0.001 | 0.397 | 0.782 |
| **Age** | -0.00 | 0.00 | -0.88 | 0.378 | -0.014 | 0.005 |
| **Education** | 0.25 | 0.09 | 2.83 | 0.005 | 0.076 | 0.421 |
| | $F(6, 489) = 7.28$, $p < 0.001$, $R^2 = 0.08$ | | | | | |
| **Pathogen Disgust (X)** | 0.23 | 0.20 | 1.10 | 0.272 | -0.177 | 0.628 |
| **PT Covid-19 (W)** | 0.13 | 0.27 | 0.49 | 0.623 | -0.392 | 0.654 |
| **Pathogen Disgust × PT Covid-19 (XW)** | -0.04 | 0.05 | -0.86 | 0.389 | -0.140 | 0.055 |
| **Sex** | 0.54 | 0.10 | 5.44 | <0.001 | 0.342 | 0.729 |
| **Age** | -0.00 | 0.00 | -0.72 | 0.747 | -0.013 | 0.006 |
| **Education** | 0.26 | 0.09 | 3.00 | 0.002 | 0.091 | 0.334 |
| | $F(6, 489) = 6.98$, $p < 0.001$, $R^2 = 0.07$ | | | | | |

*95% CI is presented as bias-corrected and accelerated 5,000 bootstrapping. Control variables: sex, age and education level PI = Perceived Infectability, GA = Germ Aversion, PT Covid-19 = Perceived Threat of Covid-19.

Table 2

*Indirect effects and confidence intervals of the Moderation Analysis of the relationship between Pathogen Disgust and homonegativity toward lesbians moderated by Perceived Infectability, Germ Aversion and Perceived Threat of Covid-19*

| | **Lesbians homonegativity** | | | | | |
|---|---|---|---|---|---|---|
| | | | | | *95% CI** | |
| | *b* | *SE* | *t* | *p* | *LL* | *UL* |
| **Pathogen Disgust (X)** | 0.11 | 0.17 | 0.66 | 0.509 | -0.225 | 0.453 |
| **Perceived Infectability (W)** | -0.04 | 0.24 | -0.16 | 0.875 | -0.509 | 0.433 |
| **Pathogen Disgust × PI (XW)** | -0.01 | 0.04 | -0.22 | 0.827 | -0.096 | 0.077 |
| **Sex** | 0.54 | 0.10 | 5.41 | < 0.001 | 0.341 | 0.734 |
| **Age** | -0.01 | 0.00 | -1.19 | 0.234 | -0.015 | 0.004 |
| **Education** | 0.25 | 0.09 | 2.83 | 0.005 | 0.077 | 0.427 |
| | $F(6, 489) = 7.69$, $p < 0.001$, $R^2 = 0.09$ | | | | | |
| **Pathogen Disgust (X)** | 0.04 | 0.18 | 0.20 | 0.841 | -0.322 | 0.395 |
| **Germ Aversion (W)** | 0.03 | 0.22 | 0.16 | 0.873 | -0.392 | 0.461 |
| **Pathogen Disgust × GA (XW)** | 0.00 | 0.04 | 0.04 | 0.966 | -0.078 | 0.081 |
| **Sex** | 0.58 | 0.10 | 5.85 | < 0.001 | 0.387 | 0.778 |
| **Age** | -0.01 | 0.00 | -1.21 | 0.227 | -0.016 | 0.004 |
| **Education** | 0.23 | 0.09 | 2.62 | 0.009 | 0.059 | 0.409 |
| | $F(6, 489) = 6.70$, $p < 0.001$, $R^2 = 0.08$ | | | | | |
| **Pathogen Disgust (X)** | 0.31 | 1.13 | 0.48 | 0.633 | -1.682 | 2.765 |
| **PT Covid-19 (W)** | 0.20 | 0.27 | 0.75 | 0.454 | -0.329 | 0.735 |
| **Pathogen Disgust × PT Covid-19 (XW)** | -0.06 | 0.05 | -1.10 | 0.273 | -0.154 | 0.044 |
| **Sex** | 0.53 | 0.10 | 5.33 | < 0.001 | 0.337 | 0.729 |
| **Age** | -0.01 | 0.00 | -1.10 | 0.270 | -0.015 | 0.004 |
| **Education** | 0.25 | 0.09 | 2.78 | 0.006 | 0.073 | 0.422 |
| | $F(6, 489) = 7.53$, $p < 0.001$, $R^2 = 0.08$ | | | | | |

*95% CI is presented as bias-corrected and accelerated 5,000 bootstrapping. Control variables: sex, age and education level PI = Perceived Infectability, GA = Germ Aversion, PT Covid-19 = Perceived Threat of Covid-19.

Table 3

*Indirect effects and confidence intervals of the Moderation Analysis of the relationship between Moral Disgust and homonegativity towards gay men moderated by Perceived Infectability, Germ Aversion and Perceived Threat of Covid-19*

| | Gay men homonegativity | | | | | |
|---|---|---|---|---|---|---|
| | | | | | *95% CI** | |
| | *b* | *SE* | *t* | *p* | *LL* | *UL* |
| **Moral Disgust (X)** | 0.12 | 0.12 | 0.94 | 0.350 | -0.128 | 0.360 |
| **Perceived Infectability (W)** | 0.02 | 0.17 | 0.14 | 0.887 | -0.306 | 0.354 |
| **Moral Disgust × PI (XW)** | -0.02 | 0.03 | -0.68 | 0.498 | -0.082 | 0.040 |
| **Sex** | 0.54 | 0.10 | 5.52 | < 0.001 | 0.346 | 0.729 |
| **Age** | -0.00 | 0.01 | -1.03 | 0.304 | -0.014 | 0.004 |
| **Education** | 0.26 | 0.09 | 3.01 | 0.003 | 0.092 | 0.435 |
| | $F(6, 489) = 8.27$, $p < 0.001$, $R^2 = 0.09$ | | | | | |
| **Moral Disgust (X)** | 0.20 | 0.19 | 1.13 | 0.260 | -0.148 | 0.553 |
| **Germ Aversion (W)** | 0.25 | 0.21 | 1.21 | 0.227 | -0.159 | 0.667 |
| **Moral Disgust × GA (XW)** | -0.04 | 0.04 | -0.96 | 0.338 | -0.112 | 0.039 |
| **Sex** | 0.59 | 0.10 | 6.07 | < 0.001 | 0.402 | 0.787 |
| **Age** | -0.00 | 0.00 | -0.99 | 0.321 | -0.014 | 0.005 |
| **Education** | 0.25 | 0.09 | 2.85 | 0.005 | 0.078 | 0.424 |
| | $F(6, 489) = 7.60$, $p < 0.001$, $R^2 = 0.09$ | | | | | |
| **Moral Disgust (X)** | 0.16 | 0.17 | 0.95 | 0.342 | -0.170 | 0.489 |
| **PT Covid-19 (W)** | 0.06 | 0.21 | 0.31 | 0.756 | -0.342 | 0.470 |
| **Moral Disgust × PT Covid-19 (XW)** | -0.03 | 0.04 | -0.75 | 0.452 | -0.106 | 0.047 |
| **Sex** | 0.53 | 0.10 | 5.40 | < 0.001 | 0.338 | 0.725 |
| **Age** | -0.00 | 0.00 | -0.87 | 0.387 | -0.013 | 0.005 |
| **Education** | 0.26 | 0.09 | 2.93 | 0.003 | 0.085 | 0.429 |
| | $F(6, 489) = 7.93$, $p < 0.001$, $R^2 = 0.09$ | | | | | |

*95% CI is presented as bias-corrected and accelerated 5,000 bootstrapping. Control variables: sex, age and education level PI = Perceived Infectability, GA = Germ Aversion, PT Covid-19 = Perceived Threat of Covid-19.

Table 4

*Indirect effects and confidence intervals of the Moderation Analysis of the relationship between Moral Disgust and homonegativity towards lesbians moderated by Perceived Infectability, Germ Aversion and Perceived Threat of Covid-19*

| | **Lesbians homonegativity** | | | | | |
|---|---|---|---|---|---|---|
| | | | | | *95% CI** | |
| | *b* | *SE* | *t* | *p* | *LL* | *UL* |
| **Moral Disgust (X)** | 0.15 | 0.13 | 1.15 | 0.249 | -0.102 | 0.394 |
| **Perceived Infectability (W)** | 0.04 | 0.17 | 0.25 | 0.807 | -0.294 | 0.377 |
| **Moral Disgust × PI (XW)** | -0.02 | 0.03 | -0.75 | 0.453 | -0.086 | 0.038 |
| **Sex** | 0.53 | 0.10 | 5.35 | < 0.001 | 0.336 | 0.725 |
| **Age** | -0.01 | 0.00 | -1.51 | 0.133 | -0.017 | 0.002 |
| **Education** | 0.25 | 0.09 | 2.76 | 0.006 | 0.071 | 0.420 |
| | $F(6, 489) = 7.61$, $p < 0.001$, $R^2 = 0.09$ | | | | | |
| **Moral Disgust (X)** | 0.21 | 0.18 | 1.14 | 0.257 | -0.150 | 0.562 |
| **Germ Aversion (W)** | 0.24 | 0.21 | 1.12 | 0.265 | -0.181 | 0.658 |
| **Moral Disgust × GA (XW)** | -0.03 | 0.04 | -0.85 | 0.394 | -0.110 | 0.043 |
| **Sex** | 0.59 | 0.10 | 5.89 | < 0.001 | 0.391 | 0.782 |
| **Age** | -0.01 | 0.00 | -1.48 | 0.141 | -0.017 | 0.002 |
| **Education** | 0.23 | 0.09 | 2.59 | 0.010 | 0.056 | 0.407 |
| | $F(6,489) = 7.05$, $p < 0.001$, $R^2 = 0.08$ | | | | | |
| **Moral Disgust (X)** | 0.20 | 0.17 | 1.19 | 0.238 | -0.133 | 0.538 |
| **PT Covid-19 (W)** | 0.10 | 0.21 | 0.49 | 0.623 | -0.309 | 0.516 |
| **Moral Disgust × PT Covid-19 (XW)** | -0.03 | 0.04 | -0.88 | 0.378 | -0.113 | 0.043 |
| **Sex** | 0.53 | 0.10 | 5.26 | < 0.001 | 0.330 | 0.724 |
| **Age** | -0.00 | 0.01 | -0.47 | 0.637 | -0.012 | 0.007 |
| **Education** | 0.24 | 0.09 | 2.68 | 0.008 | 0.064 | 0.413 |
| | $F(6, 489) = 7.23$, $p < 0.001$, $R^2 = 0.08$ | | | | | |

*95% CI is presented as bias-corrected and accelerated 5,000 bootstrapping. Control variables: sex, age and education level PI = Perceived Infectability, GA = Germ Aversion, PT Covid-19 = Perceived Threat of Covid-19.

Table 5

*Indirect effects and confidence intervals of the Moderation Analysis of the relationship between Sexual Disgust and homonegativity towards gay men moderated by Perceived Infectability, Germ Aversion and Perceived threat of Covid-19*

| | Gay men homonegativity | | | | | |
|---|---|---|---|---|---|---|
| | | | | | *95% CI** | |
| | *b* | *SE* | *t* | *p* | *LL* | *UL* |
| **Sexual Disgust (X)** | 0.06 | 0.11 | 0.54 | 0.592 | -0.151 | 0.265 |
| **Perceived Infectability (W)** | -0.32 | 0.10 | -3.20 | 0.001 | -0.511 | -0.123 |
| **Sexual Disgust × PI (XW)** | 0.06 | 0.03 | 2.27 | 0.024 | 0.008 | 0.114 |
| **Sex** | 0.78 | 0.09 | 8.35 | < 0.001 | 0.596 | 0.963 |
| **Age** | -0.00 | 0.00 | -0.15 | 0.884 | -0.009 | 0.008 |
| **Education** | 0.19 | 0.08 | 2.37 | 0.018 | 0.033 | 0.351 |
| | $F(6, 489) = 24.91, p < 0.001, R^2 = 0.23$ | | | | | |
| **Sexual Disgust (X)** | 0.11 | 0.14 | 0.82 | 0.049 | -0.158 | 0.382 |
| **Germ Aversion (W)** | -0.13 | 0.11 | -1.17 | 0.243 | -0.341 | 0.086 |
| **Sexual Disgust × GA (XW)** | 0.04 | 0.03 | 1.26 | 0.209 | -0.021 | 0.094 |
| **Sex** | 0.80 | 0.10 | 8.46 | < 0.001 | 0.617 | 0.990 |
| **Age** | 0.00 | 0.00 | -0.00 | 0.999 | -0.009 | 0.009 |
| **Education** | 0.17 | 0.08 | 2.06 | 0.040 | 0.008 | 0.330 |
| | $F(6, 489) = 21.53, p < 0.001, R^2 = 0.21$ | | | | | |
| **Sexual Disgust (X)** | 0.11 | 0.13 | 0.88 | 0.379 | -0.142 | 0.373 |
| **PT Covid-19 (W)** | -0.25 | 0.11 | -2.26 | 0.024 | -0.473 | -0.033 |
| **Sexual Disgust × PT Covid-19 (XW)** | 0.04 | 0.03 | 1.35 | 0.177 | -0.019 | 0.101 |
| **Sex** | 0.76 | 0.09 | 8.00 | < 0.001 | 0.573 | 0.946 |
| **Age** | 0.00 | 0.00 | 0.09 | 0.928 | -0.008 | 0.009 |
| **Education** | 0.17 | 0.08 | 2.14 | 0.033 | 0.014 | 0.333 |
| | $F(6, 489) = 23.41, p < 0.001, R^2 = 0.22$ | | | | | |

*95% CI is presented as bias-corrected and accelerated 5,000 bootstrapping. Control variables: sex, age and education level PI = Perceived Infectability, GA = Germ Aversion, PT Covid-19 = Perceived Threat of Covid-19.

Table 6

*Indirect effects and confidence intervals of the Moderation Analysis of the relationship between Sexual Disgust and homonegativity towards lesbians moderated by Perceived Infectability, Germ Aversion and Perceived threat of Covid-19*

| | **Lesbians homonegativity** | | | | | |
|---|---|---|---|---|---|---|
| | | | | | *95% CI** | |
| | *b* | *SE* | *t* | *p* | *LL* | *UL* |
| **Sexual Disgust (X)** | 0.08 | 0.11 | 0.73 | 0.469 | -0.134 | 0.290 |
| **Perceived Infectability (W)** | -0.30 | 0.10 | -2.97 | 0.003 | -0.496 | -0.101 |
| **Sexual Disgust × PI (XW)** | 0.06 | 0.03 | 2.08 | 0.038 | 0.003 | 0.110 |
| **Sex** | 0.78 | 0.10 | 8.18 | < 0.001 | 0.591 | 0.965 |
| **Age** | -0.00 | 0.00 | -0.59 | 0.552 | -0.011 | 0.006 |
| **Education** | 0.18 | 0.08 | 2.10 | 0.037 | 0.011 | 0.335 |
| | $F(6, 489) = 23.42$, $p < 0.001$, $R^2 = 0.22$ | | | | | |
| **Sexual Disgust (X)** | 0.13 | 0.14 | 0.95 | 0.342 | -0.142 | 0.407 |
| **Germ Aversion (W)** | -0.11 | 0.11 | -1.03 | 0.303 | -0.331 | 0.103 |
| **Sexual Disgust × GA (XW)** | 0.03 | 0.03 | 1.13 | 0.260 | -0.025 | 0.092 |
| **Sex** | 0.80 | 0.10 | 8.30 | < 0.001 | 0.612 | 0.991 |
| **Age** | -0.00 | 0.00 | -0.45 | 0.650 | -0.011 | 0.007 |
| **Education** | 0.15 | 0.08 | 1.81 | 0.070 | -0.013 | 0.315 |
| | $F(6, 489) = 20.73$, $p < 0.001$, $R^2 = 0.20$ | | | | | |
| **Sexual Disgust (X)** | 0.11 | 0.13 | 0.85 | 0.399 | -0.149 | 0.375 |
| **PT Covid-19 (W)** | -0.25 | 0.11 | -2.19 | 0.029 | -0.474 | -0.026 |
| **Sexual Disgust × PT Covid-19 (XW)** | 0.04 | 0.03 | 1.39 | 0.166 | -0.018 | 0.104 |
| **Sex** | 0.76 | 0.10 | 7.86 | < 0.001 | 0.570 | 0.950 |
| **Age** | -0.00 | 0.00 | -0.39 | 0.700 | -0.011 | 0.007 |
| **Education** | 0.16 | 0.08 | 1.88 | 0.060 | -0.007 | 0.318 |
| | $F(6, 489) = 22.32$, $p < 0.001$, $R^2 = 0.22$ | | | | | |

*95% CI is presented as bias-corrected and accelerated 5,000 bootstrapping. Control variables: sex, age and education level PI = Perceived Infectability, GA = Germ Aversion, PT Covid-19 = Perceived Threat of Covid-19.

Below, we present descriptions of supplementary effects of the covariates in moderation analyses of the relationship between sexual disgust and negative attitudes towards gay men and lesbians. In all analyses, we controlled for age (calculated as a continuous variable) and education (as a 3-categorial ordinal variable: primary, secondary and higher education).

**The Moderating Role of Perceived Infectability in the Relationship Between Sexual Disgust and Negative Attitudes Towards Gay Men**

We found a significant effect of sex, *b* = 0.78; 95% *CI* = [0.596, 0.963], which indicated that men held more negative attitudes towards gay men than women when we controlled for perceived infectability. Additionally, the education effect was significant, *b* = 0.19; 95% *CI* = [0.033, 0.351], indicating that as education increased, the negative attitudes towards gay men also increased. There was no significant effect of age, *b* = -0.00; 95% *CI* = [-0.009, 0.008].

**The Moderating Role of Perceived Infectability in the Relationship Between Sexual Disgust and Negative Attitudes Towards Lesbians**

We found a significant effect of sex, *b* = 0.78; 95% *CI* = [0.590, 0.965], which indicated that men held more negative attitudes towards lesbians than women when we controlled for perceived infectability. The education effect was also significant, *b* = 0.17; 95% *CI* = [0.011, 0.335], indicating that as education increased, negative attitudes towards lesbians also increased. The age effect was not significant, *b* = -0.00; 95% *CI* = [-0.011, 0.006].

**The Moderating Role of Germ Aversion in the Relationship Between Sexual Disgust and Negative Attitudes Towards Gay Men**

The sex effect was significant, *b* = 0.80; 95% *CI* = [0.617, 0.990], so men held more negative attitudes towards gay men than women when we controlled for germ aversion. The education effect was also

significant, $b = 0.17$; 95% $CI = [0.008, 0.330]$, indicating that as education increased, negative attitudes towards gay men also increased. We found no effect of age, $b = 0.17$; 95% $CI = [0.008, 0.330]$.

### The Moderating Role of Germ Aversion in the Relationship Between Sexual Disgust and Negative Attitudes Towards Lesbians

When we controlled for germ aversion, we found a significant effect of sex, $b = 0.80$, 95% $CI = [0.612, 0.991]$, so men held more negative attitudes towards lesbians than women. Neither education effect, $b = 0.15$, 95% $CI = [-0.013, 0.315]$, nor age effect $b = -0.00$, 95% $CI = [-0.011, 0.007]$ were significant.

### The Moderating Role of the Perceived Threat of COVID-19 in the Relationship Between Sexual Disgust and Negative Attitudes Towards Gay Men

We found a significant effect of sex, $b = 0.76$, 95% $CI = [0.573, 0.946]$, indicating that men held more negative attitudes towards gay men than women when the perceived threat of being infected by COVID-19 was a moderator. The education effect was also significant, $b = 0.17$, 95% $CI = [0.014, 0.333]$, and indicated that as education increased, negativity towards gay men also increased. There was no effect of age, $b = 0.00$, 95% $CI = [-0.001, 0.009]$.

### The Moderating Role of the Perceived Threat of COVID-19 in the Relationship Between Sexual Disgust and Negative Attitudes Towards Lesbians

We found a significant effect of sex, $b = 0.76$, 95% $CI = [0.570, 0.950]$, indicating that men held more negative attitudes towards lesbians than women when we controlled for the perceived threat of COVID-19. Neither the effect of age, $b = -0.00$, 95% $CI = [-0.011, 0.007]$, nor education, $b = 0.76$, 95% $CI = [-0.007, 0.318]$ were significant.
